# The Dose-Dependent Immunoregulatory Effects of the Nitric Oxide Synthase Inhibitor N^G^-Nitro-L-Arginine Methyl Ester in Rats with Sub-Acute Peritonitis

**DOI:** 10.1371/journal.pone.0042467

**Published:** 2012-08-03

**Authors:** Chien-Chou Hsiao, Chien-Hsing Lee, Lon-Yen Tsao, Hui-Chen Lo

**Affiliations:** 1 Department of Pediatrics, Clinical Nutrition Support Service Team, Changhua Christian Hospital, Changhau, Taiwan; 2 Division of Pediatric Surgery, Changhua Christian Hospital, Changhau, Taiwan; 3 Graduate Institute of Medical Sciences, Chang Jung Christian University, Tainan, Taiwan; 4 Department of Nutritional Science, Fu Jen Catholic University, New Taipei City, Taiwan; University of Cincinnati, United States of America

## Abstract

**Background:**

Chronic inflammation accompanied by arginine deficiency, immune dysfunction, and excess nitric oxide (NO) production is a clinical condition found in patients with peritonitis. A previous study showed that the nonselective NOS inhibitor N^G^-nitro-L-arginine methyl ester (L-NAME) may facilitate the metabolism of the immune nutrient arginine without altering NO homeostasis in rats with sub-acute peritonitis. Here, we investigated the effects of L-NAME on the immunocytic subpopulation distribution and response.

**Materials and Methods:**

Male Wistar rats with cecal puncture-induced peritonitis were administered parenteral nutrition solutions supplemented with 0 (CPP group), 5 (LNA group), 25 (MNA group) or 50 (HNA group) mg·kg^−1^·day^−1^ of L-NAME for 7 days. Parenteral-fed sham-operated rats (TPN group) and orally-fed healthy rats (R group) were included as controls.

**Results:**

The TPN group had significantly increased spleen weights and levels of plasma nitrite/nitrate (NOx), circulating white blood cells (WBC), and splenocytic T cells, as well as significantly decreased levels of cytotoxic T- and B-leukocytes and B-splenocytes compared to the R group. The CPP group had significantly decreased levels of plasma NOx and concanavalin (Con) A-stimulated interferon (IFN)-γ and interleukin (IL)-2 production by leukocytes and significantly increased production of Con A-stimulated tumor necrosis factor (TNF)-α and lipopolysaccharide (LPS)-stimulated IFN-γ in the leukocytes. In addition, the LNA and MNA groups had significantly decreased spontaneous IL-6 and Con A-stimulated TNF-α and IFN-γ production by the leukocytes while the HNA group had significantly increased LPS-stimulated TNF-α and Con A-stimulated IFN-γ and IL-2 production by the splenocytes compared to the CPP group.

**Conclusions:**

Low-dose L-NAME infusion may suppress proinflammatory and T-helper-1 (Th1) response in leukocytes, and high-dose infusion may activate the proinflammatory response in splenic macrophages and Th1 response in T-splenocytes in rats with sub-acute peritonitis.

## Introduction

Peritonitis has been considered as an alternative arginine-deficient status with abnormal immunity and altered secretion of various inflammatory mediators, such as cytokines and nitric oxide (NO), by immunocytes derived from different tissues and organs [1]. Several studies showed that arginine supplementation might improve survival and enhance the immune response [2], whereas there is considerable debate regarding arginine use in sepsis [3], [4]. Recently, we found that parenteral arginine supplementation at a dose of 2 to 6% of total calories may decrease circulating levels of interleukin (IL)-2 and nitrite/nitrate (NOx), the indirect biomarkers of NO, and may modulate the immunocytic subpopulation and cytokine production in peripheral blood leukocytes and splenocytes in a U-shaped dose-dependent manner in rats with sub-acute peritonitis [5], [6]. These inconsistent results may be associated with the activity of nitric oxide synthase (NOS) because circulating NOx concentrations are closely related to the severity of infection and sepsis [7]. Therefore, it has been proposed that the inhibition of NOS may be a useful strategy to treat arginine deficiency and to inhibit excess NO production in inflammation [8], [9].

NO is known as a regulator of inflammation and immunity and is considered as a pro-inflammatory mediator in a variety of abnormal situations. For example, NO acts as an important defense molecule against infectious organisms and regulates the activity and the growth and death of macrophages, T lymphocytes, and other immune cells. It has been demonstrated that patients with peritonitis have uncontrolled activation of inducible NOS, which results in NO overproduction and subsequent sepsis [8]. The effect of NOS inhibition on the immune response is still unclear. N^G^-nitro-L-arginine methyl ester (L-NAME) is a nonselective NOS inhibitor, which needs to be hydrolyzed by esterases to become a fully functional inhibitor of constitutive and inducible NOS. It has been reported that the administration of L-NAME may effectively ameliorate inflammatory lesions in the skin of zinc-deficient rats [10], attenuate lipopolysaccharide (LPS)-induced peritoneal permeability and NO release in mice [11], decrease oxidative stress by preserving glutathione in the brain of septic rats provoked by cecal ligation and puncture [12], and produce antidepressant-like activity through the adrenergic system and L-arginine-NO-cGMP pathway [13]. However, some studies have shown that L-NAME may reduce systemic and renal arginine turnover and increase renal protein breakdown [14], cause hypertension and augment the production of interferon (IFN)-γ and IL-2, and result in severe disease in rats with T cell-dependent autoimmune interstitial nephritis [15]. Recent evidence suggests that non-vasoactive inhibition of L-NAME is beneficial in the suppression of oxidative injury, whereas strong vasoactive inhibition of L-NAME exacerbates ischemia-reperfusion injury in rat hearts [16]. These results suggest that L-NAME has dual effects on mechanical function and energy metabolism, depending on its concentration. However, the optimal dosing of L-NAME for improving the inflammatory response is not known.

Using rats with cecal puncture-induced nonlethal peritonitis, we previously found that chronic infusion of L-NAME up to 50 mg·kg^−1^·day^−1^ may not alter circulating NOx and cytokines and may facilitate the production of arginine-associated amino acids, such as ornithine, glutamate, and proline [17]. Ornithine, a non-protein amino acid forming part of the urea cycle, can be further metabolized to polyamines, i.e., putrescine, spermidine, and spermine, to participate in lymphocyte mitogenesis [18], [19]. However, a recent study showed that increased levels of the polyamine spermine might impair immunity to *Helicobacter pylori* by inhibiting arginine uptake required for NO production [20]. It is unclear whether L-NAME-elevated plasma ornithine and unaltered circulating NOx implies an impaired immune response. Therefore, the aim of this study was to investigate the dose effects of chronic L-NAME infusion on the immunocytic subpopulation distribution and response in rats with sub-acute peritonitis.

## Materials and Methods

### Animals and Experimental Design

Male Wistar rats (8 weeks old) initially weighing approximately 220 g were supplied by the Laboratory Animal Center of the National Taiwan University, Taipei, Taiwan. All rats were housed in individual stainless steel cages with free access to water and a chow diet (1320 Rat & Mouse Maintenance diet, Altromin GmbH, Germany) in a room maintained at 22°C on a 12∶12-h light-dark cycle. The animals were acclimatized to the animal facility for 7 days before surgery. This study was carried out in strict accordance with the recommendations in the Guide for the Care and Use of Laboratory Animals of the Changhua Christian Hospital. The animal facilities and protocols were reviewed and approved by the Institutional Animal Care and Use Committee (IACUC) of the Changhua Christian Hospital, Changhua, Taiwan, with approval number CCH-AE-92008.

After the rats were fasted overnight, an intramuscular injection of ketamine (100 mg·kg^−1^ of body weight) and xylazine (10 mg·kg^−1^ of body weight) was used to perform anesthetization (day 0) to minimize suffering. Forty unconscious rats then received catheterization of the superior vena cava by way of the external jugular vein for the infusion of the parenteral nutrition solution. Subsequently, 32 rats received a second surgery for the induction of sub-acute peritonitis by a modified cecal puncture procedure [21], and the remaining eight rats received sham operations (TPN group). Eight healthy rats that had not received surgeries and had free access to water and rat chows were included as references (R group).

Animals with sub-acute peritonitis were divided into 4 groups (n = 8 per group) to receive conventional parenteral nutrition solutions supplemented with 0, 5, 25, or 50 mg·kg^−1^·day^−1^ of L-NAME for 7 days [17]. The study design was as follows:

R group: Healthy rats that received no surgery and had free access to water and rat chows.

TPN group: Sham-operated rats infused with conventional parenteral nutrition solution.

CPP group: Sub-acute peritonitic rats infused with 0 mg·kg^−1^·day^−1^ of L-NAME in the parenteral nutrition solution.

LNA group: Sub-acute peritonitic rats infused with 5 mg·kg^−1^·day^−1^ of L-NAME (low dose) in the parenteral nutrition solution.

MNA group: Sub-acute peritonitic rats infused with 25 mg·kg^−1^·day^−1^ of L-NAME (medium dose) in the parenteral nutrition solution.

HNA group: Sub-acute peritonitic rats infused with 50 mg·kg^−1^·day^−1^ of L-NAME (high dose) in the parenteral nutrition solution.

After surgery, parenteral nutrition solutions were initially infused at a slow rate and drinking water was provided *ad libitum,* but the rats were deprived of chow diet. During the experimental period, parenteral nutrition solutions provided the sole source of nutrition, and the infusion rate was gradually increased from 25 kcal on day 0 to approximately 65 kcal, i.e., 270 kcal·kg^−1^·day^−1^, on days 1 to 6. All of the rats were provided with adequate parenteral energy and nutrients for healthy rats of this size [22].

### Composition of TPN Solutions

All of the parenteral nutrition solutions were composed of crystalline amino acids, dextrose, lipid emulsion, vitamins, trace elements, and electrolytes. Each liter contained 42 g of amino acids (Aminosyn 10%, Abbott Laboratories, North Chicago, IL, USA), 160 g of dextrose (Paren-aid 50%, Taita No. V, Taiwan), and 34.6 g of lipid emulsion (20% soybean oil emulsion, Lipovenos, Fresenius AG, D-6380 Bad Homburg v.d.H. Fed. Rep. of Germany). The freshly prepared L-NAME solution was sterilized using a 0.22-µm disposable sterile filtration apparatus and added to the dextrose-amino acid mixture daily before mixing with the lipid emulsion.

### Analytical Measurements

During the experimental period, the body weights of the rats were recorded daily. At 7 days of parenteral nutrition infusion, 100% of the animals survived, i.e., 8 rats per group. On day 7, the rats were euthanized under anesthesia with intramuscular injections of 100 mg ketamine and 10 mg xylazine per kg of body weight. Blood samples were collected by cardiac puncture and divided into whole blood and plasma for further assays. The abdomen was incised for spleen collection. After weighing, the spleen was kept in ice-cold RPMI-1640 cell culture medium, which contained penicillin (100 IU/ml), streptomycin (100 µg/ml), 10 mM HEPES, 10% fetal bovine serum (FBS), and 2 mM glutamine, for splenocyte suspension.

The number of circulating white blood cells (WBCs) was determined using a hematology analyzer (GEN; Coulter Inc., Miami, FL, USA). A commercial colorimetric kit (Cayman Chemical, Ann Arbor, MI, USA) was used to determine plasma concentrations of NOx, estimated by nitrite and nitrate levels. Plasma concentrations of tumor necrosis factor (TNF)-α, IFN-γ, IL-2, IL-6, and nitrotyrosine, the indirect marker of peroxynitrite that causes tissue damage [23], were measured using enzyme-linked immunosorbent assays (ELISA; DuoSet, R&D System, Minneapolis, MN, USA; Bethyl Laboratories, Inc., Montgomery, TX, USA; and OXIS International Inc., Foster City, CA, USA). The assays for NOx, nitrotyrosine, and cytokines contained all samples in each assay and were duplicated with inter-assay coefficients of variance within 5%∼10%.

#### Immunocyte suspension

To obtain leukocytes, 4 ml of the blood sample was lysed using NH_4_Cl lysis buffer (Sigma Chemical Co.), washed twice in phosphate buffered saline (PBS) with 2% FBS, and then resuspended in RPMI 1640 medium. The single-cell suspensions of the spleen were prepared by teasing and grinding the tissue apart and flushing the cells through a 100-µm mesh screen to remove capsular material and cellular debris. Red blood cells were lysed from the splenocytes using NH_4_Cl lysis buffer and washed twice in PBS with 2% FBS to obtain single-cell suspensions. Subsequently, the complete cell counts of the single cell suspensions were quantified using a hemocytometer following trypan blue staining, and the samples were kept on ice for further assays.

#### Immunocytic subset determination

Fifty microliters of EDTA-containing whole blood was added to tubes with antibodies directed against appropriate cell-surface antigens, as reported by Morris and Komocsar [24], and incubated at room temperature for 15 min in the dark. The cell-surface markers included CD3 (clusters of differentiation)-fluorescein isothiocyanate (FITC, clone G4.18), CD3-phycoerythrin (PE, clone G4.18), CD4-PE (clone OX-35), and CD8b-FITC (clone 341) for T cells; CD45RA-PE (clone OX-33) plus IgM-FITC (clone G53–238) for B cells; and CD11b/c-PE (clone OX-42) for monocytes. FITC-conjugated mouse IgG_1_ (clone A112-2) and PE-conjugated mouse IgG_3_ (clone A112-3) were used as nonspecific isotype-control antibodies. Following incubation, red blood cells were lysed using NH_4_Cl lysis buffer (Sigma Chemical Co.), washed twice in phosphate buffered saline (PBS) with 2% FBS, and resuspended in 500 µl PBS with 1% paraformaldehyde and 0.1% NaN_3_ for flow cytometric analysis. For splenocytes, 5×10^6^ cells were added to tubes containing antibodies directed against cell-surface antigens. The antibodies were the same as those listed for the leukocyte analyses, except that CD11b/c-PE (clone OX-42) was used to label macrophages and dendritic cells. Immunofluorescent detection of cell subsets of leukocytes and splenocytes was performed using a Becton-Dickinson FACS Scan flow cytometer. To simultaneously measure the FITC- and PE-conjugated mouse anti-rat monoclonal antibodies, 488-nm (blue) laser excitation was used (BD Biosciences, San Jose, CA, USA).

#### Splenocytic proliferation

*In vitro* cell proliferation of splenocytes in response to concanavalin A (Con A, a T-cell mitogen) and polysaccharide (LPS, a B-cell and macrophage mitogen) were determined. The splenocytes were resuspended at 5× 10^6^ cells per ml of RPMI 1640 medium; 50 µl per well was plated in triplicate with 50 µl of medium with or without Con A (5 µl per ml of medium) and LPS (10 µl per ml of medium); cells were incubated at 37°C in 5% CO_2_ for 36 hours. Splenocytic proliferation was determined using the MTS method (CellTiter 96® AQ_ueous_ one solution, Promega, Madison, WI, USA). The stimulation index of splenocytic proliferation was calculated using the absorbance at 490 nm by splenocytes cultured in RPMI 1640 medium and mitogens, i.e., Con A and LPS, divided by the absorbance of those cultured in RPMI 1640 medium alone and then multiplied by 100. The unit of stimulation index was expressed as a per cent (%).

#### Cytokine production

The production of cytokines, such as TNF-α, IFN-γ, IL-2, and IL-6, by the leukocytes and splenocytes was measured by ELISA as previously described. Leukocytes and splenocytes (5×10^6^ cells per ml) were cultured with Con A (5 µg per ml of medium) and LPS (10 µg per ml of medium) at 37°C in 5% CO2 for 18 hours. The supernatants of the cultures were removed by centrifugation and stored at −80°C for further assays. All supernatants were used to measure each cytokine in one assay with duplication. The inter-assay coefficients of variance were within 10%.

### Statistical Analysis

The values were expressed as means ± SEM. All treatment groups were compared using one-way analysis of variance (ANOVA) in the SAS general linear models program. A P value less than 0.05 was considered significantly different among groups. The protective least-significant difference (LSD) technique was used for *post hoc* analysis to compare the differences between groups when the ANOVA indicated an overall significant treatment effect.

## Results

### Body Weights and Parenteral Nutrition Infusion

Animals on parenteral nutrition, i.e., the TPN, CPP, LNA, MNA, and HNA groups, gained less weight in 7 days (4.3±2.4 g) than the animals that were fed the chow diet, i.e., the R group (51.6±4.6 g). There were no significant differences in body weight and weight gain during the experimental period among the groups with sub-acute peritonitis (data not shown). In addition, the volumes of parenteral nutrition infused were 23 to 27 ml on day 1 and 60 to 63 ml on days 2 to 7 and were not significantly different among the parenteral-fed groups.

### Plasma Substrate Concentrations

Plasma concentrations of TNF-α, IL-6, IFN-γ, IL-2, NOx, and nitrotyrosine were quantified to assess systemic inflammatory responses in sub-acute peritonitic rats (Table 1). There were no significant differences in the plasma concentrations of IL-6, IFN-γ, IL-2, and nitrotyrosine among the groups. The plasma TNF-α concentration increased approximately 40 to 50% in the MNA and HNA groups compared to the R group. In addition, the plasma NOx concentration was increased 3-fold in the TPN group compared to the R group and was significantly decreased in the CPP, LNA, MNA, and HNA groups compared to the TPN group. L-NAME supplementation did not have a significant impact on plasma cytokines, NOx, and nitrotyrosine.

**Table 1:** Plasma concentrations of cytokines, nitrite/nitrate and nitrotyrosine^1^.

Group	R	TPN	CPP	LNA	MNA	HNA
**TNF-**α (µg/l)	61.7±5.4	64.1±3.6	77.9±4.0	80.9±9.2	88.5±7.6*	93.0±7.1*
**IL-6** (µg/l)	186.8±10.5	185.0±10.1	158.4±7.3	187.2±5.6	167.4±5.7	171.3±9
**IFN-**γ (µg/l)	24.9±3.3	30.2±3.5	28.4±4.4	34.5±4.7	24.1±3.5	27.5±3.8
**IL-2** (µg/l)	53.9±10.7	48.1±5.3	34.1±4.6	50.3±6.7	43.7±7.9	50.1±6.1
**NOx** (µmol/l)	66.8±4.9	242.2±18.2*	186.6±7.9*^†^	201.4±14.1*^†^	153.0±16.0*^†^	176.9±16.7*^†^
**Nitrotyrosine** (nmol/l)	2.03±0.20	3.34±0.41	1.93±0.18	2.39±1.06	2.22±0.38	2.48±0.52

1 Values are means ± SEM, n = 8 in each group. TNF, tumor-necrosis factor; IFN, interferon; IL, interleukin; NOx, nitrite/nitrate. Values with the symbols * or † are significantly different from the R and TPN groups, respectively (one-way ANOVA with least significant difference, *P*<.05).

### Spleen Weights and Numbers and Subpopulations of Immunocytes

**Figure 1:**
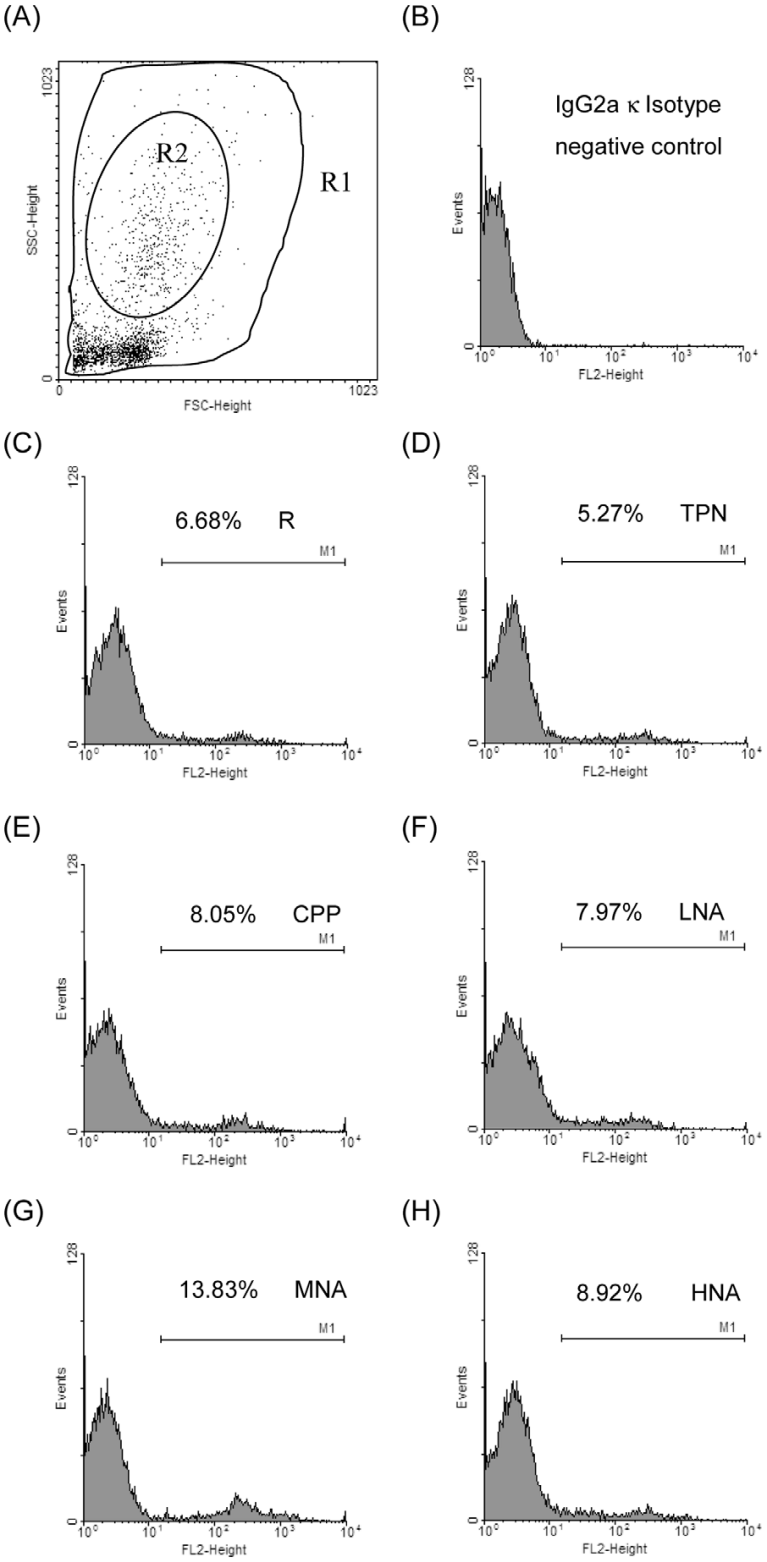
Dot plot and histograms of leukocytes labeled with CD 11b/c antibody in a FACS measurement. (A) Dot plot of forward scatter (FSC) versus side scatter (SSC) with regions R1 (almost all of the leukocytes) and R2 (granulocytes); (B) IgG2aκ isotype negative control matched FITC-labeled CD 11b/c; (C) to (H) histograms of CD 11b/c+ cells in region R1 minus region R2, i.e., monocytes in the R, TPN, CPP, LNA, MNA, and HNA groups, respectively. The numbers represent the percentages of CD 11b/c+ monocytes in leukocytes.

The numbers of circulating WBCs in the TPN group and the spleen weight and splenocytic numbers in the TPN, CPP, LNA, MNA, and HNA groups were significantly increased compared to the R group (Table 2). There were no significant differences in the percentages of total (CD3+) and helper (CD3+CD4+) T-leukocytes and helper and cytotoxic (CD3+CD8b+) T-splenocytes and splenic macrophages and dendritic cells (CD11b/c+) among the groups. Parenteral nutrition infusion significantly decreased the percentages of cytotoxic T-leukocytes, B-leukocytes (CD45RA+IgM+), and B-splenocytes and significantly increased the percentages of total T-splenocytes. The effects of L-NAME administration were observed in monocytes (CD11b/c+, Table 2 and Figure 1); the MNA group (Figure 1G) had an increase of approximately 30 to 40% in the percentages of monocytes compared to the R (Figure 1C), TPN (Figure 1D), and CPP (Figure 1E) groups.

**Table 2:** Numbers of white blood cells and splenocytes, spleen weights, and percentages of peripheral leukocytes and splenocytes^1^.

Group		R	TPN	CPP	LNA	MNA	HNA
**WBC** (10^3^/µl)		6.84±0.81	12.13±1.26*	9.23±0.66	9.32±0.81	7.86±0.64	9.77±1.36
**Spleen weight** (g)		0.69±0.03	1.46±0.05*	1.69±0.08*	1.62±0.09*	1.54±0.07*	1.47±0.06*
**Splenocytes** (×10^7^ cells)		4.44±0.65	9.76±3.31*	9.08±1.69*	11.57±3.07*	8.57±1.45*	9.17±2.70*
**Leukocytes** (%)	CD3^+^	47.6±3.7	46.8±2.0	48.3±2.6	48.4±3.5	45.9±0.7	47.2±1.2
	CD3^+^CD4^+^	32.1±1.9	34.9±1.8	36.1±3.6	37.3±4.7	37.1±0.5	36.5±0.9
	CD3^+^CD8b^+^	11.57±1.28	7.81±0.77*	8.85±0.73*	8.41±1.09*	7.00±0.19*	8.61±0.43*
	CD45RA^+^IgM^+^	12.23±2.37	6.81±0.87*	5.23±0.59*	5.89±1.03*	4.74±0.43*	6.06±0.67**
	CD11b/c^+^	6.75±0.59	6.32±0.72	8.44±0.57	7.91±1.10	11.34±0.61*^†§^	8.55±0.36^†^
**Splenocytes** (%)	CD3^+^	32.6±0.2	41.4±2.0*	41.9±2.0*	43.4±3.3*	42.3±1.3*	41.6±0.6*
	CD3^+^CD4^+^	17.3±0.8	19.1±0.2	20.5±0.6	20.1±1.2	21.1±1.3	20.8±0.5
	CD3^+^CD8b^+^	8.78±0.63	11.64±1.02	12.16±1.27	12.91±1.81	13.28±0.52	13.66±1.02
	CD45RA^+^IgM^+^	28.0±0.8	16.2±1.4*	16.4±1.3*	14.8±1.2*	15.6±1.0*	16.0±0.6*
	CD11b/c^+^	12.49±0.90	11.26±1.85	9.52±0.50	9.08±0.70	10.44±0.34	9.07±0.47

1 Values are means ± SEM, n = 8 in each group. WBC, white blood cells; CD3+, total T cells; CD3+CD4+, helper T cells; CD3+CD8b+, cytotoxic T cells; CD45RA+IgM+, B cells; CD11b/c+, monocytes/macrophages and dendritic cells. Values with the symbols *, ^†^, or ^§^ are significantly different from the R, TPN, and CPP groups, respectively (one-way ANOVA with least significant difference, *P*<.05).

### Splenocyte Proliferation

**Figure 2:**
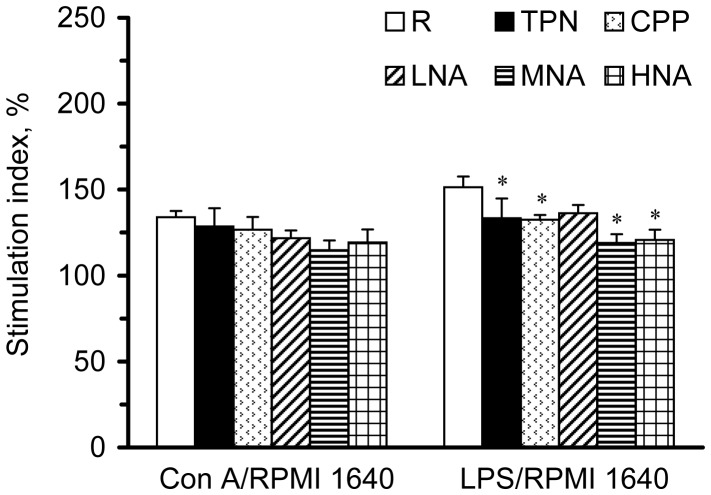
Stimulation indices of cell proliferation in splenocytes cultured in medium, Con A, and LPS. The stimulation index was calculated from OD values (at 490 nm) of splenocytes cultured with Con A or LPS divided by those in RPMI 1640 medium and multiplied by 100. Values with the symbol * are significantly different from the R group (one-way ANOVA with least significant difference, P<.05).

To evaluate the response of the splenocytes to mitogen stimulation, we measured their proliferative capacity with and without stimulation by Con A and LPS. The optical density (OD) values for the splenocytes cultured in RPMI 1640 medium were not significantly different among the groups (data not shown). When calculated as stimulation indices, Con A-stimulated proliferation in the splenocytes was not significantly different among the groups (Figure 2). However, parenteral nutrition infusion significantly decreased LPS-stimulated proliferation. Neither sub-acute peritonitis nor L-NAME administration had significant impacts on Con A- or LPS-stimulated proliferation in the splenocytes.

### Cytokine Production of Leukocytes

**Figure 3:**
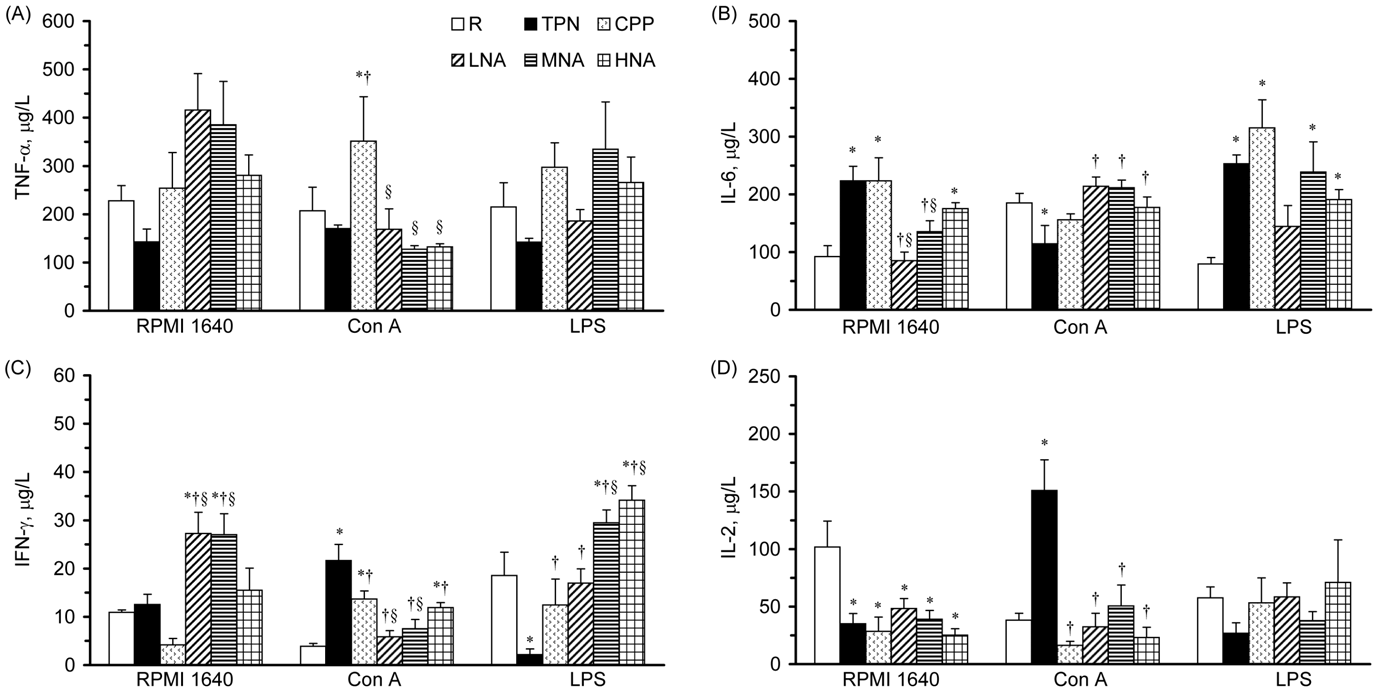
TNF-α (A), IL-6 (B), IFN-γ (C), and IL-2 (D) production by leukocytes (5×10^6^ cells) cultured in medium with or without Con A (5 µg/ml) and LPS (10 µg/ml). Values are means ± SEM, n = 8. TNF, tumor-necrosis factor; IFN, interferon; IL, interleukin. Values with the symbols *, †, or § are significantly different from the R, TPN, and CPP groups, respectively (one-way ANOVA with least significant difference, P<.05).

The results of cytokine production from leukocytes with or without mitogen stimulation are shown in Figure 3. TNF-α production by leukocytes without mitogen stimulation (i.e., spontaneous production) and with LPS stimulation was not significantly different among groups, whereas that with Con A stimulation was significantly increased in the CPP group compared to the TPN and R groups and was significantly decreased in the LNA, MNA, and HNA groups compared to the CPP group (Figure 3A). IL-6 production by leukocytes without mitogen stimulation was significantly increased in the TPN group compared to the R group and was significantly decreased in the LNA and MNA groups compared to the TPN and CPP groups (Figure 3B). However, IL-6 production by leukocytes in the TPN group was significantly decreased with Con A stimulation and was significantly increased with LPS stimulation compared to the R group. The Con A-stimulated IL-6 production was significantly increased in the LNA, MNA, and HNA groups compared to the TPN group.

The spontaneous production of IFN-γ by leukocytes was significantly increased in the LNA and MNA groups compared to the R, TPN, and CPP groups (Figure 3C). When stimulated with Con A, IFN-γ production was significantly increased in the TPN group compared to the R group, decreased in the CPP group compared to the TPN group, and further decreased in the LNA and MNA groups compared to the CPP group. In contrast, when stimulated with LPS, IFN-γ production by leukocytes was significantly decreased in the TPN group compared to the R group, increased in the CPP group compared to the TPN group, and further increased in the MNA and HNA groups compared to the CPP group.

The spontaneous production of IL-2 by leukocytes was significantly decreased in the TPN group compared to the R group (Figure 3D). The Con A-stimulated production of IL-2 was significantly increased in the TPN group and significantly decreased in the CPP group compared to the R and TPN groups, respectively. There were no significant differences in LPS-stimulated IL-2 production among the groups. L-NAME administration did not have a significant impact on spontaneous and Con A- or LPS-stimulated production of IL-2 in leukocytes.

### Cytokine Production of Splenocytes

**Figure 4:**
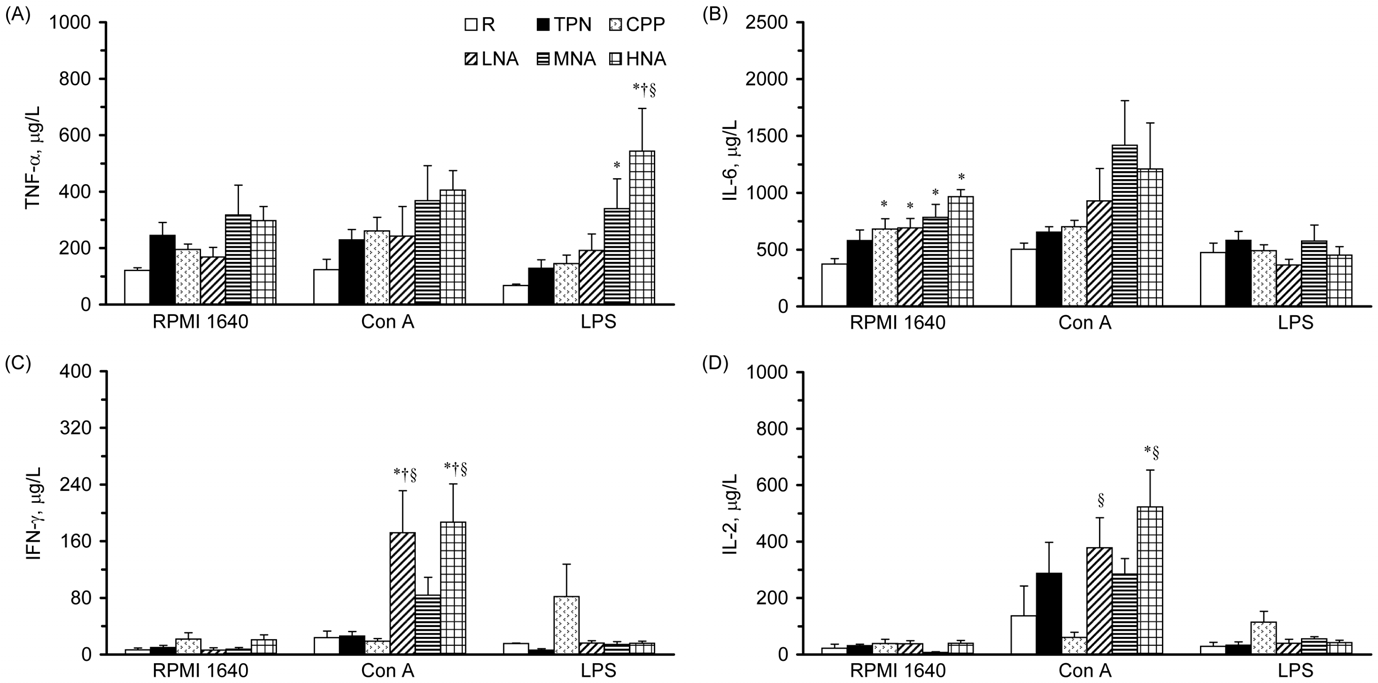
TNF-α (A), IL-6 (B), IFN-γ (C), and IL-2 (D) production by splenocytes (5×10^6^ cells) cultured in medium with or without Con A (5 µg/ml) and LPS (10 µg/ml). Values are means ± SEM, n = 8. TNF, tumor-necrosis factor; IFN, interferon; IL, interleukin. Values with the symbols *, †, or § are significantly different from the R, TPN, and CPP groups, respectively (one-way ANOVA with least significant difference, P<.05).

For the cytokine production of splenocytes (Figure 4), there were no significant differences in spontaneous and Con A-stimulated TNF-α production among groups; however, LPS-stimulated TNF-α production was significantly increased in the HNA group compared to the R, TPN, and CPP groups (Figure 4A). In addition, parenteral nutrition infusion, sub-acute peritonitis, and L-NAME administration did not have a significant impact on spontaneous, Con A-stimulated, and LPS-stimulated IL-6 production (Figure 4B). There were no significant differences in the spontaneous or the LPS-simulated production of IFN-γ and IL-2 in splenocytes among the groups (Figure 4C and 4D). However, the LNA and HNA groups had significantly increased LPS-stimulated production of IFN-γ and IL-2 in splenocytes compared to the CPP group.

## Discussion

Immune dysfunction is a clinical problem in patients with chronic peritonitis, which is usually accompanied by a state of immune-paralysis presenting as a diminished response of pro-inflammatory cytokines in macrophages and progressive increases in NO and CD8+ T cells in the peritoneum [5], [25]. In rats with sub-acute peritonitis, an appropriate dose of parenteral arginine, a well-known immunonutrient, may enhance leukocytic and splenocytic responses [6], and continuous infusion of L-NAME (25 mg·kg^−1^·day^−1^) may increase arginine-associated metabolites without altering the circulating NO status or inducing significant adverse responses [17]. In the present study, continuous infusion of 5 and 25 mg·kg^−1^·day^−1^of L-NAME significantly decreased TNF-α and IFN-γ production by T-leukocytes and 50 mg·kg^−1^·day^−1^of L-NAME significantly increased TNF-α production by splenic macrophages and IFN-γ and IL-2 production by T-splenocytes in rats with sub-acute peritonitis. These results suggest that the immunoregulatory effects of L-NAME on the immunocytic response are dose-dependent in sub-acute peritonitis.

Total parenteral nutrition infusion is commonly associated with gut atrophy, bacterial translocation, and gut-associated lymphoid tissue degeneration, resulting in immune alteration [26]. In rats with an 80% small bowel resection, a 3-day parenteral nutrition infusion significantly increased the circulating numbers of WBCs, spleen weights, and T-splenocytes and significantly decreased the number of macrophages in the spleen [27]. Moreover, it has been demonstrated that parenteral feeding may elevate epithelial cell apoptosis in the intestine and may alter the expression of cytokine mRNA and decrease the numbers of total, CD4+ (helper), and CD8+ (suppressor) T cells in intraepithelial lymphocytes in mice with parenteral nutrition compared to those with enteral nutrition for 7 days [28]. In the present study, we found similar effects of parenteral nutrition on WBCs and T-splenocytes in rats with sub-acute peritonitis. In addition, parenteral-fed rats had significantly decreased levels of circulating T-cytotoxic leukocytes, B-leukocytes, and B-splenocytes (Table 1) and declined splenocytic B/macrophage proliferation (Figure 2). These results revealed that parenteral nutrition infusion might decrease both T and B lymphocytes in the circulation and spleen, as well as perhaps decrease splenocytic proliferation.

The literature indicates that TNF-α and IL-6 are among the most important cytokines produced by monocytes and macrophages in the innate immune system and that T-helper (Th) 1 and Th2 cytokines produced by helper-T cells play a key and differential role in regulating the innate immune response of monocytes [29]. For example, IFN-γ induces and IL-4 blocks autophagy by monocytes. In the present study, parenteral-fed rats had significantly elevated production of pro-inflammatory cytokines, i.e., IL-6 (Figure 3C), in monocytes and significantly declined productions of IL-6 in T-leukocytes. In contrast, the productions of Th1 cytokines, i.e., IL-2 and IFN-γ (Figure 3B and 3D), were significantly increased in T-leukocytes and decreased in the monocytes. These findings suggested that parenteral nutrition might activate the innate immune response of monocytes via elevated Th1 cytokines derived from T-leukocytes.

It is well known that changes in the immune response play a decisive role in clinical outcomes during inflammation and sepsis. The elevated systemic production of inflammatory cytokines such as IL-6, TNF-α, and IFN-γ may result in a “cytokine storm” that leads to significant end-organ damage and death [30]. In contrast, the concomitant production of anti-inflammatory cytokines may balance the inflammatory state. Muenzer et al. [31] observed that septic mice with cecal ligation and punctures had a significant loss in lymphocytes, a significant increase in neutrophils, and a significant decrease in the ability of splenocytes to produce IFN-γ. In the current study, parenteral-fed rats with nonlethal, sub-acute peritonitis (i.e., the CPP group) did not show significant changes in the plasma concentrations of TNF-α, IL-6, IFN-γ, and IL-2, the numbers of circulating WBCs and splenocytes, or the subpopulations of leukocytes and splenocytes compared to parenteral-fed rats with a sham operation (i.e., the TPN group). The major changes appeared to be the cytokine production of immunocytes. We believe that the differences between these two models may occur because rats with nonlethal, sub-acute peritonitis suffered a milder stress compared to mice with cecal ligation and punctures, as supported by the survival rates of 100% and 40% after 4 days of insults, respectively. It is also possible that the immunosuppressive effects of parenteral feeding concealed the immune defects caused by peritonitis in this model.

The importance of NO and arginine in the immune response has been evaluated in many studies. NO, a short half-life free radical derived from arginine, has been implicated in the pathophysiology of acute peritonitis for its ability in regulating the migration of neutrophils and cytokine synthesis and the bacterial killing of macrophages [32], [33]. In an endotoxin-induced septic rat model, Boughton-Smith and colleagues found that inhibition of NOS by 12.5 to 50 mg⋅kg^−1^ of L-NAME might dose-dependently reduce the increases in colonic and jejunal vascular permeability against endotoxin damage [34]. They suggested that early inhibition of NOS, i.e., the first 4 hours of LPS injection, may inhibit cNOS and exacerbate injury, whereas the late inhibition of NOS may inhibit iNOS and function in a protective manner. To date, no study has investigated the dose effects of L-NAME infusion on immune response.

Using sub-acute peritonitic rats, we found that a medium dose of L-NAME (i.e., 25 mg·kg^−1^·day^−1^) significantly increased monocytic subsets (Table 1). In addition, animals with low and medium doses (i.e., 10 and 25 mg·kg^−1^·day^−1^, respectively) of L-NAME had significantly decreased spontaneous IL-6 production and Con A-stimulated TNF-α and IFN-γ production and significantly increased spontaneous IFN-γ production by leukocytes (Figure 3). Moreover, medium and high doses (i.e., 25 and 50 mg·kg^−1^·day^−1^, respectively) of L-NAME significantly increased LPS-stimulated IFN-γ production in the leukocytes. It has been suggested that circulating monocytes may produce more anti-inflammatory mediators to prevent an uncontrolled systemic immune response against the infected tissues [18]. Although we did not determine the production of anti-inflammatory cytokines in immunocytes, the elevated Th1 response of monocytes and the diminished pro-inflammatory and Th1 responses of T-leukocytes implied that parenteral L-NAME may increase innate immunity and decrease adaptive immunity in sub-acute peritonitis in a dose-dependent manner. According to the results of our previous study, sub-acute peritonitic animals with chronic inhibition of L-NAME (≤ 25 mg·kg^−1^·day^−1^) had significantly increased plasma arginine and ornithine [17], an arginase-catalyzed metabolite and a precursor of polyamines and proline, without affecting systemic NO homeostasis (Table 2). Therefore, we speculated that the elevated circulating arginine and ornithine, two amino acids with immunoregulatory activities, might be associated with the changes in the immunocytic response [35].

The immunocytic response of the spleen, a representative systemic lymphoid organ, was used to evaluate the effects of parenteral L-NAME on the immune response in rats with sub-acute peritonitis. We found that even though the splenocytic proliferation with Con A and LPS stimulation was not significantly altered by parenteral feeding, sub-acute peritonitis, or parenteral L-NAME administration, the production of Th1 cytokines, i.e., IFN-γ and IL-2, by T-splenocytes was significantly increased in rats infused with L-NAME compared to those without L-NAME (Figure 4). In addition, rats treated with a high dose of L-NAME had a significantly increased production of pro-inflammatory cytokine, such as TNF-α from macrophages in the spleen. The elevated Th1 and proinflammatory cytokines from mitogen-stimulated splenocytes indicated that a low dose of L-NAME may activate adaptive immunity and a high dose of L-NAME may activate both adaptive and innate immunities in the spleen in sub-acute peritonitis.

Our previous studies showed that the parenteral-fed, sub-acute peritonitic rats gained less body weight and had a lower carcass protein content and serum albumin concentration. They also had increased liver and spleen weights accompanied by sacs composed of muscular and connective tissue filled with intra-abdominal abscesses [17], [21]. Moreover, the circulating numbers of WBCs were significantly increased. All of these symptoms indicate that these peritonitic animals were under catabolic and inflammatory stress, even though their circulating levels of TNF-α and NOx were not significantly increased. In addition, the doses we used were lower than those used in other rat studies, for example, 300 mg/kg^−1^ [36]. The lower dose, continuous infusion of L-NAME should be safe for rats. Taken together, our findings suggest that patients with parenteral nutrition and sub-acute inflammation may benefit from chronic L-NAME infusion to normalize the immune response. In addition, the dose of the L-NAME infusion should depend on the immune status of patients.

There are several limitations regarding this study that are worth noting. First, we did not collect peritoneal inflammatory cells to analyze the cytokine profiles in the abdominal cavity to evaluate the local inflammatory and immune responses. Second, we only obtained samples on day 7 to investigate the chronic effects of L-NAME infusion. Therefore, we might have lost track of the immediate effects of L-NAME infusion on immunity. Third, we did not include an orally-fed group with sub-acute peritonitis to distinguish the effects of parenteral nutrition solution via enteral and parenteral feedings on immunity. Therefore, the experimental design of the current study can only describe the immunoregulatory effects of L-NAME in sub-acute peritonitic rats with parenteral feeding. However, we think that the current study design is more practical and may mimic the patho-physiological responses of hollow-organ perforation found in patients with non-lethal peritonitis and parenteral nutrition support.

In the present study, several techniques could be improved to truly reflect the immune functions, for example, using intracellular protein antibodies with different CD markers (i.e., T cells or monocytes) to determine which types of cells are proliferating and determining intracellular cytokines by flow cytometry to understand the immune function of T cells and monocytes/macrophages. The effects of anesthetic agents on the immune response may also be considered. For example, the combination of ketamine and xylazine has been shown to attenuate LPS-induced upregulation of iNOS in the spleen 6 hours after injection [37]. Because the rats in this study were killed within 5 minutes of anesthetization and all of the rats were treated in the same way, the effects of ketamine and xylazine on the immune response might be negligible.

In summary, the results of this study confirmed that parenteral nutrition may result in immune dysregulation, as shown by the decreased leukocytes and splenocytes and altered immunocytic subpopulations and cytokine production in leukocytes. In addition, parenteral-fed rats with sub-acute peritonitis had activated proinflammatory and diminished Th1 responses in leukocytes. When chronically infused with a low dose of L-NAME, the sub-acute peritonitis-activated proinflammatory response was attenuated and the diminished Th1 responses were further decreased in leukocytes, suggesting a mitigated inflammatory response. However, a high dose of L-NAME may elevate the Th1 response in T-splenocytes and increase the proinflammatory response in splenic macrophages in rats with sub-acute peritonitis. In conclusion, chronic infusion of L-NAME may modulate adaptive and innate immunities in a dose- and tissue-dependent manner. These findings provide evidence that L-NAME infusion may modulate parenteral feeding and peritonitis-associated immune alterations and that the dose should depend on the immune profiles of the subjects. More studies are needed to elucidate the regulatory mechanism of L-NAME.
